# RAISN: Robot-assisted Indocyanine Green–guided Sentinel Node Biopsy in Clinical Stage I Germ Cell Tumor

**DOI:** 10.1016/j.euros.2024.06.004

**Published:** 2024-06-27

**Authors:** Marieke Sofie Vermeulen-Spohn, Pailin Pongratanakul, Sophia Thy, Juergen Dukart, Peter Albers, Yue Che

**Affiliations:** a Department of Urology, University Hospital Düsseldorf, Heinrich Heine University Düsseldorf, Düsseldorf, Germany; b Institute of Neurosciences and Medicine, Forschungszentrum Jülich, Jülich, Germany

**Keywords:** Testicular cancer, Nodal staging, Occult metastasis, Sentinel lymph nodes, Lymph node biopsy, Retroperitoneal lymphadenectomy, Indocyanine green

## Abstract

Robot-assisted imaging-guided sentinel lymph node biopsy is a novel technique that has not been widely investigated in testicular germ cell tumor (GCT). Current staging strategies have poor accuracy for prediction of occult metastatic disease in clinical stage I GCT. Feasibility studies have used ^99m^Tc-nanocolloid staining during laparoscopic procedures. The RAISN trial is investigating robot-assisted lymph node resection guided by indocyanine green fluorescence imaging. This new diagnostic approach is potentially more precise and easier to apply, and is widely available. Confirmation of its utility could change the management of newly diagnosed GCT by reducing overtreatment and treatment-related toxicity.

## Take Home Message

The RAISN trial is investigating the sensitivity of robot-assisted sentinel lymph node biopsy using free indocyanine green for detection of occult metastatic disease in patients with newly diagnosed testicular cancer.

## 1 Introduction

Most patients with a diagnosis of testicular cancer initially present with clinical stage I disease [1]. Among these patients, depending on testicular histology, 20–50% experience recurrence during active surveillance because of occult metastatic disease [2]. Although the overall long-term survival for patients with testicular cancer is very favorable, chemotherapy- and radiotherapy-induced long-term toxicity is associated with a higher incidence of side effects such as cardiovascular events and secondary malignancies [3], [4], [5]. Risk-adapted strategies that are based on histological findings for the primary specimen have been adopted to reduce the overall burden of adjuvant treatment. However, these strategies have low sensitivity and specificity and still lead to overtreatment in 50–70% of patients receiving adjuvant chemotherapy [2]. Therefore, there is a need to improve diagnostic procedures to ultimately reduce the risk of progression from occult metastatic disease while minimizing overtreatment.

Pilot studies investigating sentinel lymph node (SLN) diagnosis using radioactive tracers in testicular cancer have shown high sensitivity (∼90%; Table 1). In 2005, Satoh et al [6] described radionuclide-labeled lymph node staging in testicular cancer via SLN biopsy. In addition to orchiectomy, the authors performed technetium-guided laparoscopic sentinel node resection. The SLN detection rate was 95%. Two patients had positive SLNs and were treated with two cycles of adjuvant bleomycin, etoposide, and cisplatin (BEP) chemotherapy. Over follow-up of 20 mo, two relapses occurred in patients who previously had negative SLN biopsies. These false-negative findings were attributed to detection error.

**Table 1:** Studies that investigated laparoscopic radioguided sentinel biopsy in testicular cancer

Study	Patients	SN detection rate (%)	Tumors detected	aCTx	Follow-up (mo)	Recurrences
Satoh 2005 [6]	22	95	2	2	20	2 (SN-negative)
Brouwer 2011 [7]	10	100	1	1	21	0
Blok 2019 [8]	27	93	3	4	64	0

aCTx = adjuvant chemotherapy; SN = sentinel node

In 2011, Brouwer et al [7] conducted a study that used single-photon emission computed tomography (SPECT)/computed tomography (CT) and a γ-camera for image-guided laparoscopic SLN biopsy in ten patients with testicular cancer. The SLN detection rate was 100%, and one SLN harbored tumor. The patient was treated with four cycles of BEP. No relapses were observed during 21 mo of follow up.

A later study by Blok et al [8] included 27 patients with stage I testicular cancer who underwent ^99^Tc-guided laparoscopic SLN biopsy using the previously published technique. The SLN detection rate was 93% and tumor-positive biopsies were observed in three patients who were successfully treated with chemotherapy. All of these studies have shown that mapping of SLNs in patients with clinical stage I testicular germ cell tumor (GCT) is feasible. Furthermore, the high negative predictive values mean that adjuvant treatment can potentially be avoided by all patients with tumor-negative SLNs. The consistent results from these studies justify further investigation of SLN staging in testicular GCT.

RAISN will be the first trial to use indocyanine green (ICG) as a nonradionuclide staining technique for SLN dissection in patients with testicular GCT. The use of free ICG has certain limitations and some advantages in comparison to radionuclide or hybrid tracers. Tc-nanocolloid or hybrid ICG-Tc-nanocolloid facilitates preoperative SLN localization using lymphoscintigraphy and SPECT/CT. For intraoperative guidance, the radionuclide emits a signal that can be traced with a γ-probe. Hybridization between the Tc-nanocolloid and ICG does not seem to alter the biological performance of the radionuclide and allows better intraoperative optical guidance thanks to ICG fluorescence [9]. Previous studies used a radionuclide tracer, and it is unknown if the same results can be expected with free ICG, although several studies in gynecological malignancies have demonstrated the noninferiority of free ICG in comparison to radioligand-guided SLN detection [10], [11]. Although a study using a hybrid ICG-Tc-nanocolloid tracer would have been feasible in our department, we consciously decided against this after thorough deliberation. First, a hybrid ICG-radioactive tracer limits greatly the workflow of the procedure. Tc-nanocolloid must be injected at least several hours before surgery and lymphoscintigraphy or SPECT/CT represents an additional diagnostic intervention. In the setting of newly diagnosed testicular cancer, for which many diagnostic procedures are performed within a short time, this would negatively impact patient recruitment. As SLN staging is not standardized in testicular cancer, there is no imperative need for a comparative study with Tc-nanocolloid. Furthermore, our aim is to develop a strategy with wide reproducibility. Almost every urology department performs laparoscopic and robotic surgery, but not every hospital has a nuclear medicine department. The use of free ICG for SLN staging has already been established in uterine cancer [12]. ICG also reliably marks SLNs during lymph node staging in gastric cancer [13] and colorectal cancer (Delphi consensus recommendation) [14].

The primary objective of the RAISN trial is to establish a precise and reliable tool for nodal staging in clinical stage I testicular GCT. Our hypothesis is that surgical escalation from inguinal orchiectomy to an additional minimally invasive SLN biopsy is associated with substantial benefits that outweigh the inherent surgical risks. By potentially reducing the need for adjuvant chemotherapy and pre-empting relapses via immediate robot-assisted retroperitoneal lymph node dissection (RPLND) for patients with positive SLNs, the RAISN strategy holds promise for reducing the need for systemic therapy. Moreover, patients with negative SLNs may opt for less burdensome surveillance protocols, alleviating the psychological strain associated with prolonged monitoring.

The RAISN study protocol was approved by the ethics committee of Düsseldorf (project number 2023-2328).

## 2 Study design

**Fig. 1:**
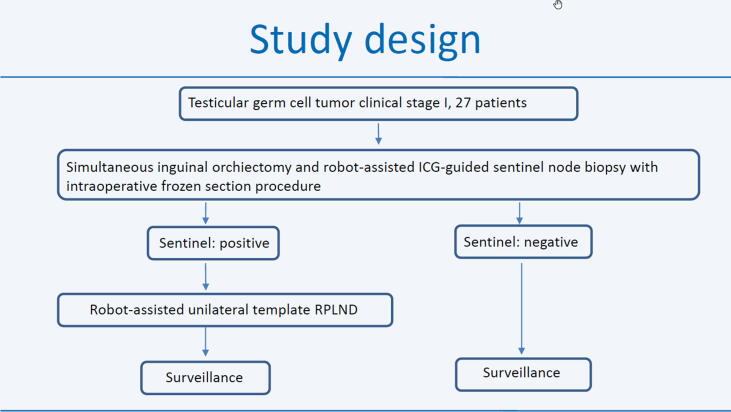
Study design.

RAISN is a single-center, phase 2, prospective clinical study (Fig. 1). Patients must fulfill the following eligibility criteria:

­ An unambiguous, clinically confirmed testicular tumor on palpation and sonography with or without elevation of the tumor markers AFP and/or β-hCG.
­ No evidence of thoracoabdominal metastasis on preoperative CT staging.
­ Age ≥18 yr.
­ The patient can communicate with the investigator without any problems or restrictions and can fulfill the study requirements.
­ The patient can understand and sign the patient declaration and consent form without any problems or restrictions.

We defined the following ineligibility criteria:

­ Previous scrotal or retroperitoneal surgery for an indication other than GCT.
­ Previous chemotherapy.
­ Previous radiotherapy to the retroperitoneum.
­ Allergy to ICG or iodine (ICG solution contains sodium iodide).
­ Low general health status or a life-threatening illness.
­ A mental illness.

The recruitment phase will last 12–24 mo and the aim is to recruit 27 patients. The first evaluation and analysis of the results will begin after 24 mo of follow-up for the last patient included. The full follow-up period will be 5 yr, resulting in an overall study duration of 7 yr. RAISN is being conducted in the Department of Urology at Düsseldorf University Hospital.

## 3 Protocol overview

**Fig. 2:**
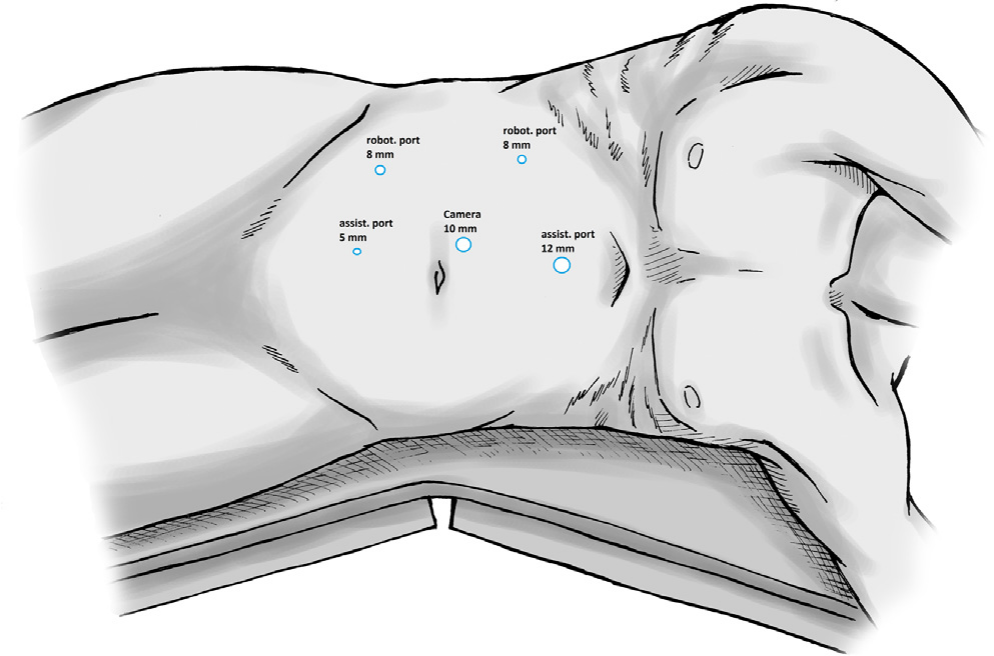
Patient positioning for surgery (right-sided).

**Fig. 3:**
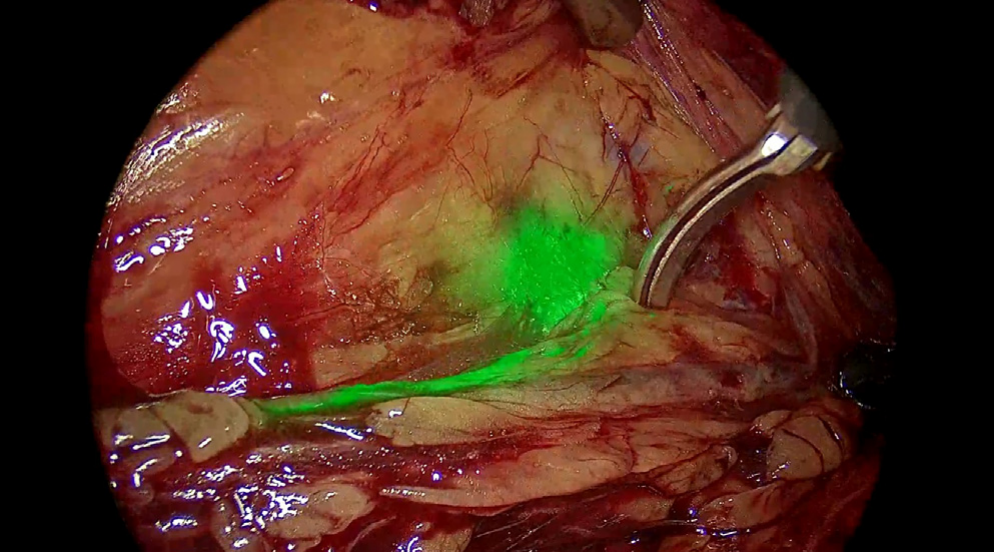
Sentinel node without fluorescence camera.

Patients with a clinically evident testicular tumor (tumor marker elevation, sonography, palpation findings) undergo preoperative thoracoabdominal CT imaging. If no metastatic disease is detected on staging CT, the patient can be included in the study. In addition to standard therapy (radical inguinal orchiectomy), study patients undergo robot-assisted fluorescence-guided SLN biopsy during the same surgery, which is performed as follows. The patient is positioned in the nephrectomy position (Fig. 2). After trocar and instrument placement, the anatomic plane between the colon and the kidney is carefully dissected and the colon is mobilized medially. At this step the posterior peritoneum is left unopened to maintain intact lymphatic pathways. ICG solution is then injected trans-scrotally into the parenchyma of the testis surrounding the tumor. The retroperitoneal space is then explored using the infrared camera, and ICG migration through the lymphatic vessels is carefully observed (Fig. 3). The first lymph node marked by the ICG fluorescence signal is deemed the SLN. If more than one lymph node is marked simultaneously, all marked lymph nodes are deemed SLNs. The harvested SLN(s) are sent for immediate frozen section evaluation. Inguinal orchiectomy is performed while awaiting results for the SLN biopsy. If the biopsy is negative, the procedure is completed. If the SLN biopsy shows vital tumor, there is evidence of clinical stage IIA and a unilateral template RPLND is performed. Before the surgery, the patient is informed about the possibility of template RPLND if a positive SLN is detected. After the procedure, all patients undergo surveillance and do not receive adjuvant systemic treatment. Serum and tissue samples are stored in a biobank for further analysis. After completion of follow-up for all patients, clinical and molecular analyses of the biobank material will be performed. The results could provide new information on risk stratification for clinical stage I GCT that could guide future treatment strategies in this setting.

The surgeon performing the intervention must have already carried out at least 20 robot-assisted RPLND procedures in patients with testicular GCT (including operations after chemotherapy) to guarantee consistent surgical quality and safety.

## 4 Sample size calculation and study endpoints

The primary study objective is to prove that RAISN has sensitivity of ≥90% in nodal staging of clinical stage I GCT for correct identification of patients who have occult metastatic disease. The sample size is a consequence of aiming for sensitivity of at least 90%, which was justified on the basis of the exceptionally high sensitivity observed in prior studies. Given that RAISN is more surgically invasive than standard inguinal orchiectomy, we decided that demonstration of very high sensitivity is crucial to justify this escalation in surgical intervention.

On the basis of epidemiological follow-up data for seminoma and nonseminoma, overall sensitivity of 73.6% was calculated for the risk-adapted approach [2] in which patients are categorized as having low or high risk, with surveillance for the low-risk group and adjuvant chemotherapy for the high-risk group.

The sample size calculation was based on the following data [15]. The prevalence of nonseminoma versus seminoma is 37% versus 63%. In the nonseminoma group, 27% of cases are classified as high risk, and 50% of these patients experience metastasis during follow-up, compared to 17% in the low-risk group. In the seminoma group, 70% are considered at high risk, and 32% of these patients experience metastasis, compared to only 12% in the low-risk group. The sample size was calculated using the OneSampleProportion.Equality option in R *TrialSize* version 1.4 (https://cran.r-project.org/web/packages/TrialSize/index.html). According to calculations, a sample size of 27 patients will demonstrate better sensitivity of at least 90% over the risk-adapted approach (sensitivity of 73.6%) with power of 80% at a significance level of α = 0.05. Sensitivity is determined on the basis of true-positive and false-negative results. All sentinel lymph nodes with tumor detection are to be evaluated as true positives. The number of false-negative results is determined as the number of patients who suffer a relapse by the 2-yr follow-up assessment. As the minimum follow-up is 2 yr, all patients will have data for this assessment.

According to reports indicating that the incidence of occult metastatic disease among patients with clinical stage I GCT is 21% [2], we anticipate that five patients will have occult metastatic disease (95% confidence interval 1.08–8.91). The low number of patients expected to have metastasis poses a challenge in calculating the sample size for the trial. Consequently, a single false-negative result will mean that the primary endpoint cannot be achieved if patients with metastasis are encountered at the anticipated number in a sample size of 27. In the event of false-negative results, we have defined the following decision rules. If a single false-negative result is observed during follow-up, the sample size will be extended to 48 patients (ten expected patients with occult metastatic disease). If two false-negative results are observed, the sample size will be extended to 96 patients. The extended sample sizes can be realistically recruited within a time frame of 2–3 yr. If three or more false-negative results occur, the trial will be stopped.

Secondary endpoints are progression-free survival and the added value of assessing prognostic markers at clinical and molecular levels (primary tumor and metastases). Further endpoints will be perioperative and long-term complications according to the European Association of Urology and Clavien-Dindo classification schemes, health-related quality of life, mental health, and the proportion of patients with postoperative retrograde ejaculation. There are no discontinuation criteria defined for the study.

## 5 Conclusions

RAISN is the first trial of ICG-guided, minimally invasive SLN staging in patients with clinical stage I testicular GCT. This new approach will potentially be able to detect occult metastasis and thus may identify patients who could avoid adjuvant treatment. The RAISN trial will evaluate the diagnostic accuracy and oncological efficacy of an SLN-based surveillance strategy in patients with clinical stage I testicular GCT.

  ***Conflicts of interest***: The authors have nothing to disclose.

  ***Funding/Support and role of the sponsor:*** The sponsor does NOT have a role in the design and conduct of the study.
